# Myoid Gonadal Stromal Tumor of the Testis: A Rare Sex Cord-Stromal Neoplasm in a Young Man

**DOI:** 10.7759/cureus.115535

**Published:** 2026-08-31

**Authors:** Ernest A Morton, Samuel R Nieder, Alexander Abdollahzadeh, Dylane Davis, Jason Sina, Patrick Mullane, Reza Lahiji, Brendan Browne

**Affiliations:** 1 Department of Urology, Emory University School of Medicine, Atlanta, USA; 2 Department of Pathology, Emory University School of Medicine, Atlanta, USA; 3 Winship Cancer Institute, Emory University, Atlanta, USA

**Keywords:** gonadal stromal tumor, immunohistochemistry, mgst, myoid gonadal stromal tumor, orchiectomy, sex cord-stromal tumor, testicular mass, testicular neoplasm, urologic oncology, urologic pathology

## Abstract

Myoid gonadal stromal tumor is a rare subtype of testicular sex cord-stromal tumor characterized by a mixture of myoid and gonadal spindle cells. We present a 24-year-old man with a two-year history of chronic, intermittent dull pain in the left testicle. Work-up, management, pathology, and follow-up are reviewed. These tumors have demonstrated indolent behavior, typically benign; however, accurate diagnosis is essential to differentiate them from other more dangerous tumor types. Radical orchiectomy alone appears to be sufficient for the treatment of these tumors, with only short-term surveillance appearing adequate due to their indolent nature.

## Introduction

Myoid gonadal stromal tumor (MGST) is a rare form of testicular sex cord-stromal tumor that has only recently been classified, characterized by spindle cell morphology with both myoid and gonadal stromal differentiation on permanent pathologic staining [1]. Testicular cancers occur in approximately six per 100,000 men annually in the United States, with 90-95% arising from the germ cell lineage responsible for spermatogenesis. In contrast, testicular sex cord-stromal tumors account for approximately 5% of testicular neoplasms and arise from, or show differentiation toward, the supportive and hormone-producing elements of the testis, including Leydig, Sertoli, granulosa, and related stromal cells [2]. Here we report the case of a young man with a testicular mass with subsequent diagnosis of MGST.

MGSTs have several histologic and immunophenotypic features in common with other sex cord-stromal tumors. However, unlike sex cord-stromal tumors, which have rare undifferentiated subtypes with aggressive behavior, MGSTs have consistently lacked features of robust mitotic activity, vascular invasion, necrosis, and atypical mitosis [3].

The initial clinical evaluation of MGST does not differ substantially from that of other testicular masses and includes physical examination, scrotal ultrasonography, and serum measurement of testicular cancer tumor markers alpha-fetoprotein (AFP), beta-human chorionic gonadotropin (β-hCG), and lactate dehydrogenase (LDH). Symptoms, tumor markers, and imaging findings are often nonspecific, where MGST may clinically and radiographically resemble a germ cell tumor or another sex cord-stromal neoplasm. Definitive distinction therefore requires the examination of permanent histologic sections with appropriate immunohistochemical staining.

Sex cord-stromal tumors have a lower metastatic potential than germ cell tumors, with Leydig cell tumors having the highest risk among sex cord-stromal tumors at ~10% [4]. Current literature indicates that MGST has minimal metastatic risk, with all cases reported to date showing benign behavior without recurrence or metastasis after surgical treatment [1,5]. Due to the excellent prognosis of MGST, it distinguishes this tumor from atypical or unclassifiable sex cord-stromal tumors [6]. Given the rarity of this tumor, it is exceedingly important to capture and report all cases in an effort to increase the understanding of the full spectrum of the disease and more confidently confirm its benign nature. The aim of this report is to illustrate the clinical presentation, pathological features, management, and follow-up considerations pertinent to MGST.

## Case presentation

A 24-year-old man with no medical history presented to the emergency department (ED) due to a chronic and intermittent dull left testicular pain that has been present for the past two years. Physical exam revealed a non-tender, firm nodule within the superior aspect of the left testicle with no associated scrotal swelling or skin changes. Genitourinary history revealed no history of cryptorchidism nor a family history of testicular cancer in any first-degree relative. A scrotal ultrasound was obtained in the ED and was significant for a 1.5 cm hypoechoic mass with increased vascularity noted within the left testis (Figure 1).

**Figure 1:**
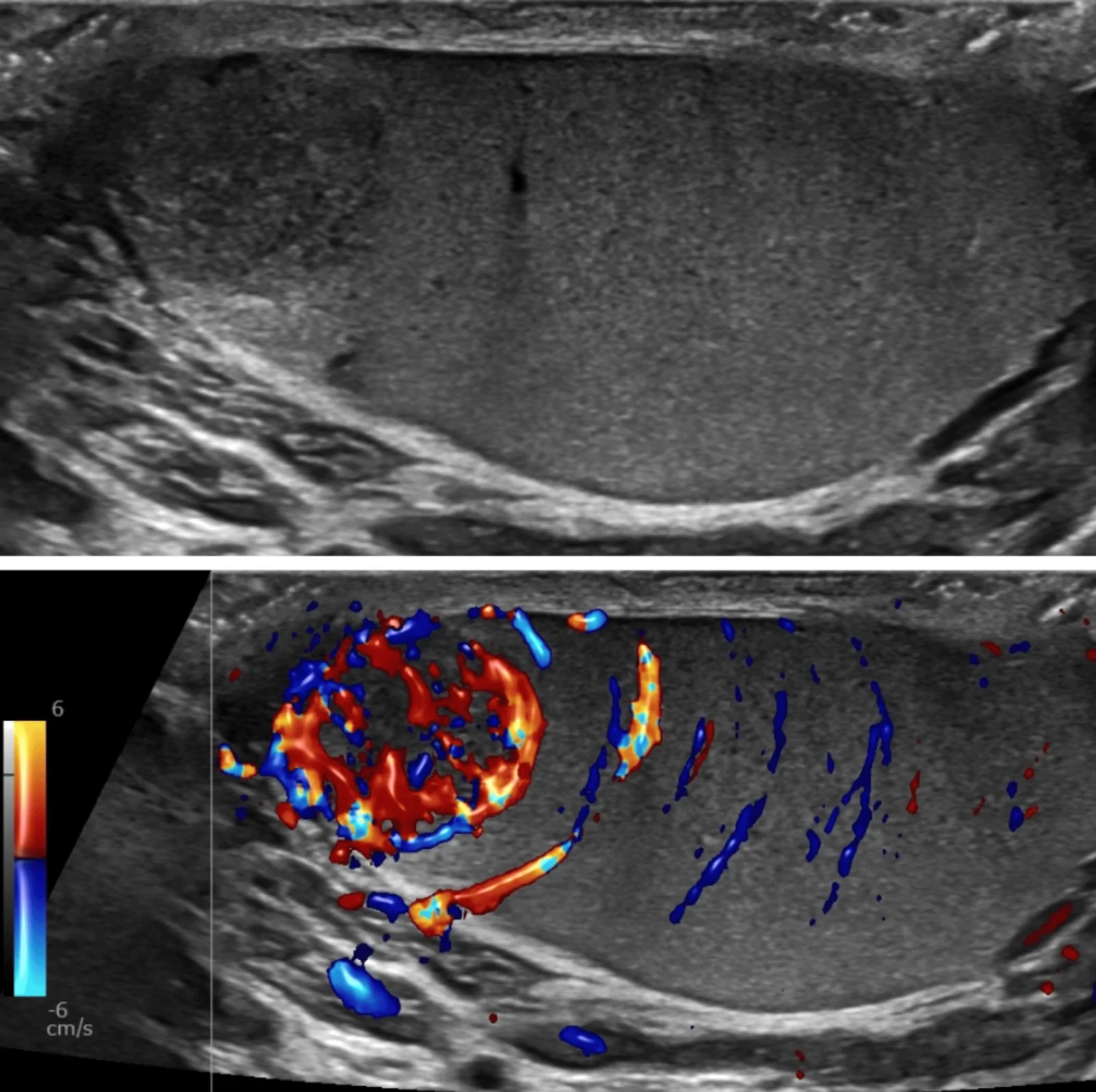
Scrotal ultrasound demonstrating a hypoechoic nodule within the superior aspect of the left testis measuring 1.5 cm in size and hypervascularity

Testis tumor markers (AFP, β-hCG, LDH) were drawn and all resulted within normal limits. He was discharged from the ED with short-term clinic follow-up. Based on ultrasound and physical exam findings, the differential diagnosis included germ cell tumors, Leydig cell tumors, or other sex cord-stromal tumors, and the decision was made to proceed with left inguinal radical orchiectomy to confirm pathology. The surgery was performed in the traditional fashion, and the patient's post-op course was uncomplicated. Pathologic diagnosis showed MGST with no germ cell neoplasia in situ, and surgical margins were negative. A gross pathology image of the testicular lesion is shown in Figure 2.

**Figure 2:**
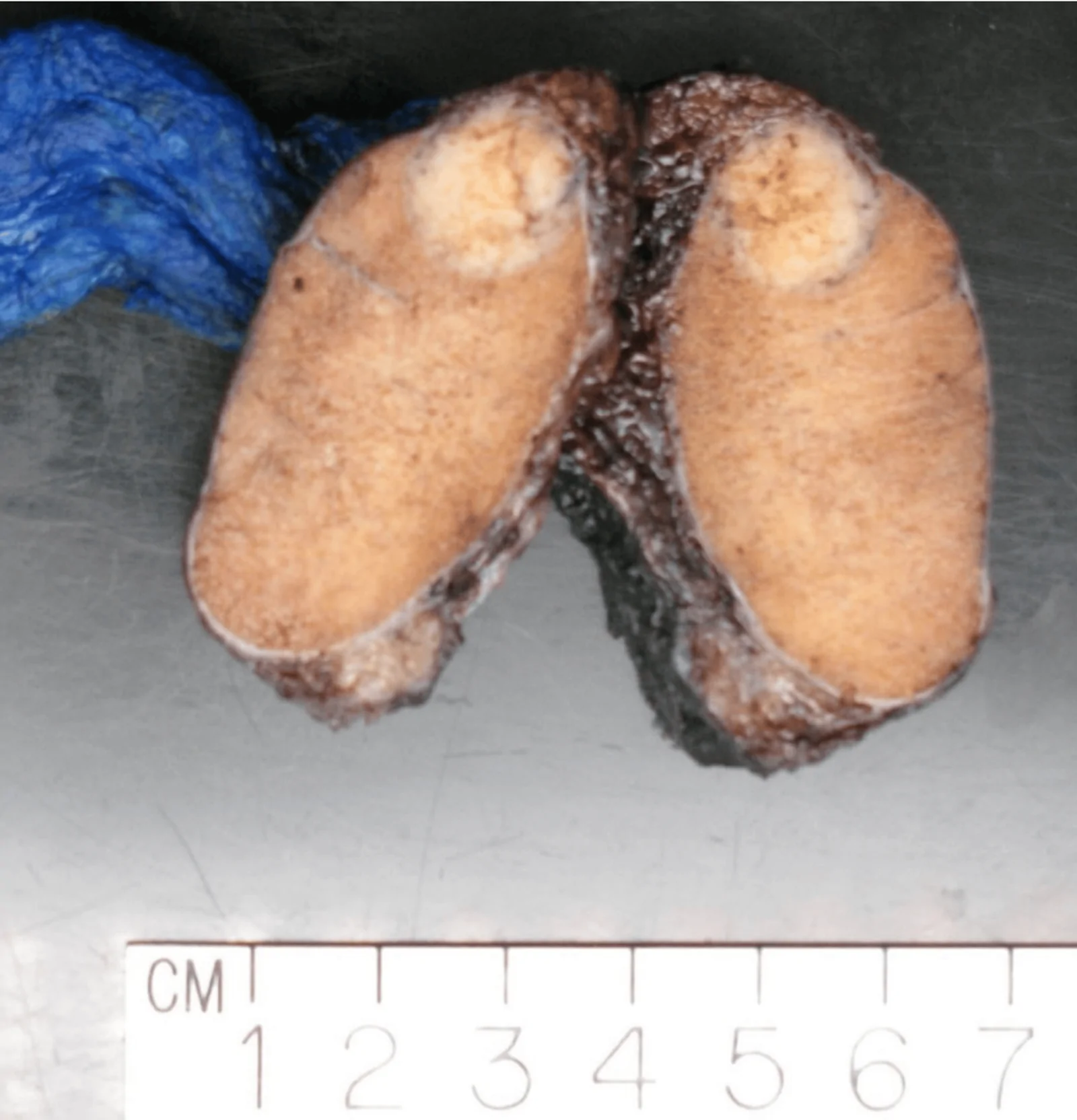
Gross pathology photo Radical orchiectomy surgical pathology specimen: a 1.5 cm unencapsulated, well-circumscribed, white-tan homogenous mass is present at the superior pole of the testis. The mass is adjacent to the rete testis and the head of the epididymis.

The patient returned to the clinic eight months postoperatively, after multiple attempts were made to arrange earlier follow-up. At that visit, a physical examination revealed a well-healed incision and a normal contralateral testicle with no firmness, induration, or palpable nodules. Surveillance imaging was also recommended, including scrotal ultrasound and CT of the abdomen and pelvis, to evaluate for abdominal masses and assess the contralateral testicle. If initial postoperative imaging was negative, no additional surveillance was to be planned. The patient did not complete the recommended imaging and has not returned to the clinic since despite numerous attempts to contact him.

## Discussion

The first published case of MGST in 1977 was in a four-year-old boy, wherein ultrastructural analysis revealed a testicular tumor containing cells rich in cytoplasmic microfilaments and dense bodies that were characteristic of smooth muscle [7]. Owing to their extremely low prevalence, these tumors were only formally recognized as a distinct entity in the 2022 edition of the WHO Classification of Tumours of the Urinary System and Male Genital Organs [8]. Formal recognition should increase diagnostic awareness and facilitate more consistent classification of future cases.

MGST is exceedingly rare, with only around 20 cases reported in the literature [9-11]. It predominantly affects younger adults, with a mean age of 40 years, and typically presents as a small, unilateral testicular mass, with a median size of approximately 20 mm [10]. Similar to sex cord-stromal tumors, tumor markers AFP, β-hCG, and LDH are typically negative. On ultrasound imaging, all the reported tumors present as small, solid, hypoechoic lesions, with vascularity. Given the similarity of the clinical presentation to other testicular lesions, specifically the more dangerous category of germ cell tumors, definitive histopathologic and immunohistochemical evaluation is necessary to determine the treatment course.

Of the 22 published cases, 20 underwent radical orchiectomy, and the other two underwent testis-sparing surgery. To date, there is no documented metastatic disease or recurrence in patients with MGST [1,9,10]. Given the favorable behavior reported to date and the rarity of MGST, no formal postoperative surveillance guidelines currently exist. Review of previously reported cases demonstrates various surveillance schedules ranging from 0 to 70 months with scrotal ultrasound noted as the most common imaging modality utilized for surveillance.

The paucity of information surrounding these tumor types can make obtaining an accurate pathologic diagnosis extremely challenging, especially at centers without a dedicated or specialized genitourinary pathologist. Considering that subsequent treatment for certain testicular masses following radical orchiectomy can include chemotherapy, radiation, or retroperitoneal lymph node dissection, obtaining an accurate diagnosis is imperative to avoid unnecessary treatment and its associated risks.

From an immunohistochemical standpoint, MGSTs are distinct from other sex cord-stromal and mesenchymal tumors of the testis. MGSTs are characterized by a neoplastic population of pure spindled cells arranged typically in short fascicles that demonstrate smooth muscle and gonadal stromal differentiation [8]. The spindled tumor cells are cytologically bland with ovoid to tapered nuclei and ill-defined, pale to lightly eosinophilic cytoplasm (Figure 3).

**Figure 3:**
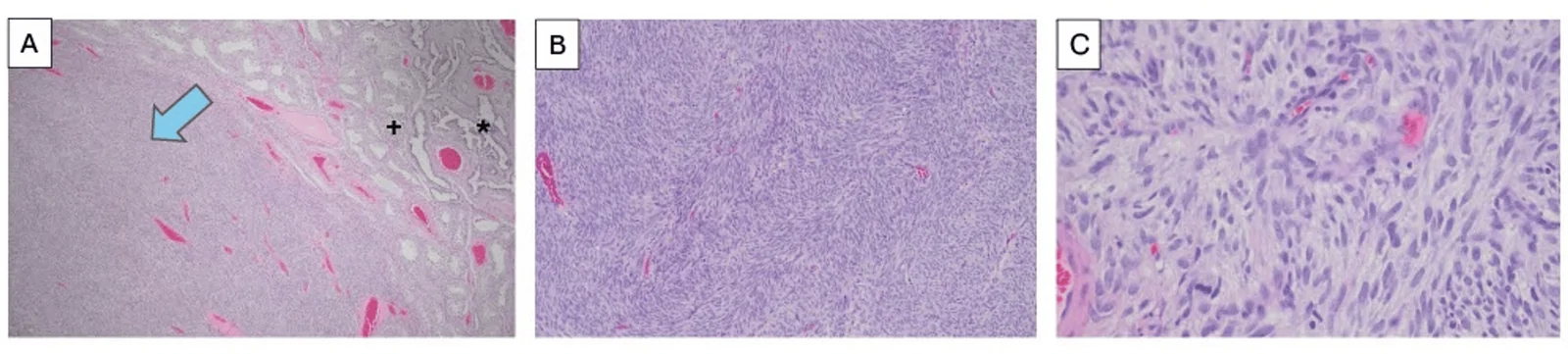
Microscopy (H&E) images (A) Low-power view shows the well-defined myoid gonadal stromal tumor (blue arrow) in relation to the rete testis (*) and background seminiferous tubules (+) (H&E, 40×). (B) Intermediate-power view shows a uniform population of tumoral spindle cells arranged in short fascicles (H&E, 100×). (C) High-power view shows fusiform spindle cells with ill-defined, pale to lightly eosinophilic cytoplasm. Occasional mitotic figures are present (H&E, 400×). H&E: hematoxylin and eosin

The tumors display a unique immunohistochemical phenotype: positive for general gonadal markers forkhead box protein L2 (FOXL2) and steroidogenic factor 1 (SF1), diffuse and strong expression of S100 and smooth muscle actin (SMA), focal positivity for inhibin, and negative staining for SRY-box transcription factor 9 (SOX9), h-caldesmon, and calretinin. Additionally, staining for reticulin fibers demonstrates preserved pericellular distribution (Figure 4).

**Figure 4:**
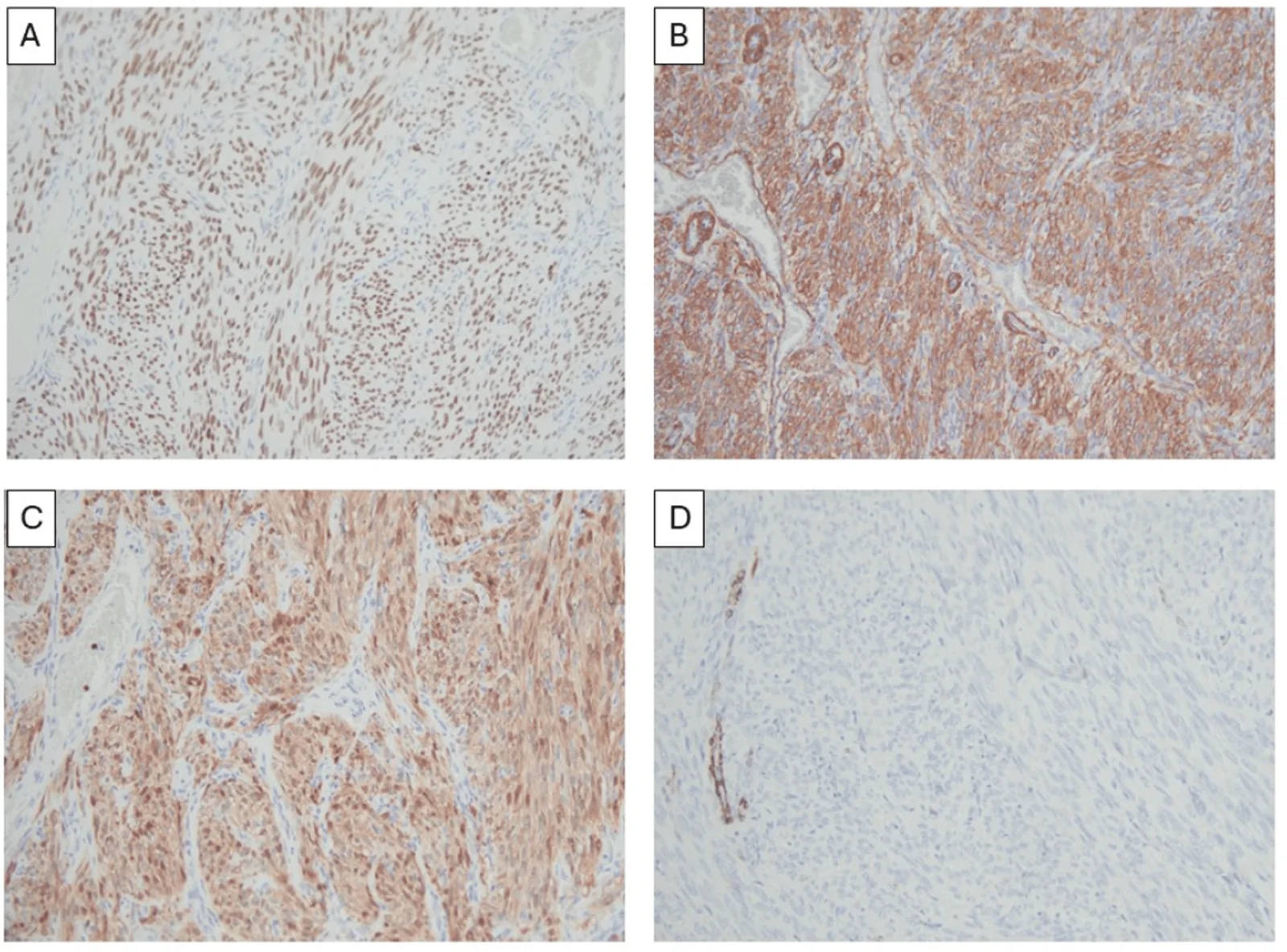
Immunohistochemistry images The tumor is strongly positive for SF1 (A, 200×), SMA (B, 200×), and S100 (C, 200×) and negative for caldesmon (D, 200×). SF1: steroidogenic factor 1; SMA: smooth muscle actin

The main differential diagnoses for MGST include sex cord-stromal tumor with incomplete or undifferentiated sex cord or stromal elements (i.e., "sex cord-stromal tumor not otherwise specified (NOS)"), leiomyoma, and testicular tumors of the fibroma thecoma group ("testicular fibrothecoma"). Unlike sex cord-stromal tumor NOS, which demonstrates at least focal sex cord differentiation characterized by a distinct population of rounder cells arranged in nests or cords with inconspicuous reticulin fibers, the neoplastic cells of MGST are purely spindled and retain their pericellular reticulin meshwork. Leiomyoma and testicular fibrothecoma may also present as histologically pure spindled cell neoplasms but demonstrate key morphologic and/or immunophenotypic differences. Leiomyomas classically are composed of cigar-shaped/box-car nuclei with abundant eosinophilic cytoplasm and occasional perinuclear halos arranged in broad, sweeping fascicles, histologic features not observed in MGSTs. Immunohistochemically, leiomyomas strongly express h-caldesmon and are negative for S100, SF1, and FOXL2, findings that are opposite those of MGST [10]. Lastly, testicular fibrothecoma, particularly cellular lesions lacking the thick collagen deposition and hyaline plaques characteristic of this entity, may show significant morphologic overlap with MGST. Immunohistochemically, both tumors show positive reactivity for the general gonadal markers SF1 and FOXL2; however, only MGSTs show strong and diffuse S100 expression. Additionally, testicular fibrothecoma is positive for inhibin and SOX9, whereas MGSTs are focally positive for inhibin and negative for SOX9 [5] (Table 1).

**Table 1:** Comparison of clinicopathologic and immunohistochemical features of the current case with previously reported MGST cases Mitotic activity is reported as mitoses per HPF. Data attributed to Elousrouti et al. [9] represent pooled clinicopathologic findings from the previously reported cases summarized in their literature review, rather than findings from their individual case. "Patchy/variable" inhibin staining indicates heterogeneity in staining among the cases included in the literature review. HPF: high-power field; SMA: smooth muscle actin; SF-1: steroidogenic factor 1; MGST: myoid gonadal stromal tumor

	Age (years)	Size (cm)	Mitosis per HPF	Immunohistochemistry					
				SMA	Inhibin	Desmin	SF-1	Calretinin	Caldesmon
Our patient	24	1.5	5	Positive	Patchy/variable	Negative	Positive	Negative	Negative
Elousrouti et al. [9]	38.5	2	1.17	Positive	Patchy/variable	Negative	Positive	Negative	Negative

Inguinal radical orchiectomy remains the standard treatment for testicular masses suspicious for malignancy and accounts for the treatment in the majority of prior cases of MGST [10,12]. Testis-sparing surgery with intraoperative frozen-section examination may be considered for selected small, marker-negative masses when the lesion is technically amenable to local excision and appropriate surgical and pathological expertise is available [12]. However, the diagnostic capacity of frozen sections depends largely on specimen quality, and limited specimens may pose difficulty for differentiating stromal tumors from the mesenchymal elements of a teratoma or a spindled yolk sac tumor. Additionally, even if a sex cord-stromal tumor is identified on a frozen section, there are rare reports of malignant behavior that should be considered when deciding on testis-sparing surgery [6]. In this patient, radical orchiectomy was selected because high-risk malignancy could not be excluded with preoperative imaging, the superior-pole location raised concern for compromise of the testicular blood supply during partial orchiectomy, and the patient favored definitive treatment.

Although surveillance is a widely accepted strategy following orchiectomy for testicular cancer, adherence to recommended follow-up protocols remains poor, and low adherence may compromise the effectiveness of this management strategy [13]. Historically, young men with testicular cancer have low levels of follow-up [14], which may be explained by the unique psychosocial profile of this patient population. Young men are less likely than older patients to acknowledge the threat of serious illness, and the loss of a gonad at an age when sexuality and body image are central to identity creates additional barriers to ongoing engagement with care [15]. Emerging strategies to address these challenges include mobile health applications and telemedicine, which have demonstrated improved adherence, high patient and clinician satisfaction, and potential cost savings by reducing the travel and time burden associated with frequent surveillance visits [13,16]. No formal postoperative surveillance guidelines exist for MGST. Because no recurrence or metastatic progression has been reported in the small volume of published cases, a limited surveillance strategy was recommended for this patient. Unfortunately, the incomplete follow-up for this patient prevents definitive conclusions regarding the optimal duration or modality of surveillance and the long-term malignant potential of MGST. Accordingly, the favorable behavior described in the literature should be interpreted cautiously until larger series with longer follow-up are available.

## Conclusions

MGSTs of the testis are exceedingly rare and may present similarly to other testicular neoplasms, including as a hypoechoic, vascular intratesticular lesion with normal serum tumor markers. Definitive diagnosis requires permanent histopathologic examination with appropriate immunohistochemical staining. Available published reports suggest favorable behavior without documented recurrence or metastasis; however, the limited number of reported cases and incomplete follow-up in the present patient preclude definitive conclusions regarding long-term malignant potential or optimal surveillance. Additional cases with longer follow-up are needed to better define management and postoperative surveillance strategies.
